# Does reservoir host mortality enhance transmission of West Nile virus?

**DOI:** 10.1186/1742-4682-4-17

**Published:** 2007-05-11

**Authors:** Ivo M Foppa, Andrew Spielman

**Affiliations:** 1 Department of Epidemiology, Arnold School of Public Health, University of South Carolina, 800 Sumter Street, Columbia, SC 29208, USA; 2 Department of Immunology and Infectious Diseases, Harvard School of Public Health, 665 Huntington Avenue, Boston, MA 02515, USA

## Abstract

**Background:**

Since its 1999 emergence in New York City, West Nile virus (WNV) has become the most important and widespread cause of mosquito-transmitted disease in North America. Its sweeping spread from the Atlantic to the Pacific coast was accompanied by widespread mortality among wild birds, especially corvids. Only sporadic avian mortality had previously been associated with this infection in the Old World. Here, we examine the possibility that reservoir host mortality may intensify transmission, both by concentrating vector mosquitoes on remaining hosts and by preventing the accumulation of "herd immunity".

**Results:**

Inspection of the Ross-Macdonald expression of the basic reproductive number (*R*_0_) suggests that this quantity may increase with reservoir host mortality. Computer simulation confirms this finding and indicates that the level of virulence is positively associated with the numbers of infectious mosquitoes by the end of the epizootic. The presence of reservoir incompetent hosts in even moderate numbers largely eliminated the transmission-enhancing effect of host mortality. Local host die-off may prevent mosquitoes to "waste" infectious blood meals on immune host and may thus facilitate perpetuation and spread of transmission.

**Conclusion:**

Under certain conditions, host mortality may enhance transmission of WNV and similarly maintained arboviruses and thus facilitate their emergence and spread. The validity of the assumptions upon which this argument is built need to be empirically examined.

## Background

In 1999, West Nile virus (WNV) emerged in North America with a massive and deadly avian epizootic in New York City [1] that was accompanied by a cluster of human meningo-encephalitis cases [2]. Among the avian species affected by that epizootic, corvids and certain exotic zoo specimens were particularly obvious [3]. Since then, WNV has disseminated across the entire contiguous United States and southern Canada, becoming the most common arboviral disease in North America. More recently, evidence of WNV transmission has been reported from Central America, South America and the Caribbean [4-7]. Although Work and colleagues, in their original studies on WNV transmission [8], observed 100% mortality in experimentally infected hooded crows (*Corvus cornix*), substantial and widespread mortality in wild birds had not previously been noted. In the year preceding the emergence of WNV in North America, however, a number of domestic geese (*Anser anser*) and white storks (*Ciconia ciconia*) died of WNV infection in Israel [9,10]. That variant of the virus was closely related to the virus introduced into North America [11]. Avian mortality became a key signature of WNV in North America, with corvids particularly severely affected [12-16].

Here, we examine the possibility that substantial reservoir host mortality might modify transmission dynamics of WNV and thus might have impacted emergence of WNV in North America. By means of a simple quantitative model, we first examine the potential impact of extreme virulence on enzootic transmission. Then, we examine the relationship between virulence and enzootic WNV transmission dynamics by the use of a stochastic individual-based computer simulation. Finally, we interpret our findings in the context of WNV ecology and epidemiology and formulate potential evolutionary implications.

## Results

### Quantitative argument

The inherent ability of an infectious agent to perpetuate is quantified in terms of its basic reproductive number, generally denoted as *R*_0 _[17]. In the simplest case of a homogeneously mixing population, *R*_0 _is defined as the average number of secondary cases deriving from each index case in an "entirely susceptible" population. Large and persistent outbreaks of an infectious agent are only possible if *R*_0 _exceeds one [18]. Therefore, *R*_0 _is crucial for the epidemiological characterization of a transmission system. The following simple expression for *R*_0 _of malaria transmission was first formulated by Macdonald [19]:

R0=m a2 d pn bm bh−ln⁡p,
 MathType@MTEF@5@5@+=feaafiart1ev1aaatCvAUfKttLearuWrP9MDH5MBPbIqV92AaeXatLxBI9gBaebbnrfifHhDYfgasaacH8akY=wiFfYdH8Gipec8Eeeu0xXdbba9frFj0=OqFfea0dXdd9vqai=hGuQ8kuc9pgc9s8qqaq=dirpe0xb9q8qiLsFr0=vr0=vr0dc8meaabaqaciaacaGaaeqabaqabeGadaaakeaacqWGsbGudaWgaaWcbaGaeGimaadabeaakiabg2da9maalaaabaGaemyBa0MaeeiiaaIaemyyae2aaWbaaSqabeaacqaIYaGmaaGccqqGGaaicqWGKbazcqqGGaaicqWGWbaCdaahaaWcbeqaaiabd6gaUbaakiabbccaGiabdkgaInaaBaaaleaacqWGTbqBaeqaaOGaeeiiaaIaemOyai2aaSbaaSqaaiabdIgaObqabaaakeaacqGHsislcyGGSbaBcqGGUbGBcqWGWbaCaaGaeiilaWcaaa@47E1@

where *m *is the mosquito-host ratio, *a *the biting rate of a female mosquito, *d *the duration of infectiousness in human hosts, *p *the daily survival probability of mosquitoes, and *n *the duration of the extrinsic incubation period in days. The extrinsic incubation period refers to the time between an infectious blood meal and infectiousness. The transmission parameters *b*_*m *_and *b*_*h *_quantify the probability of transmission from an infectious mosquito to a susceptible person and from an infectious person back to a mosquito, respectively. Because of Sir Ronald Ross's conceptual contribution [20], this expression is often designated as the "Ross-Macdonald expression". The Ross-Macdonald expression can be factored into two terms,

m a d bh×pn a bm−ln⁡p.
 MathType@MTEF@5@5@+=feaafiart1ev1aaatCvAUfKttLearuWrP9MDH5MBPbIqV92AaeXatLxBI9gBaebbnrfifHhDYfgasaacH8akY=wiFfYdH8Gipec8Eeeu0xXdbba9frFj0=OqFfea0dXdd9vqai=hGuQ8kuc9pgc9s8qqaq=dirpe0xb9q8qiLsFr0=vr0=vr0dc8meaabaqaciaacaGaaeqabaqabeGadaaakeaacqWGTbqBcqqGGaaicqWGHbqycqqGGaaicqWGKbazcqqGGaaicqWGIbGydaWgaaWcbaGaemiAaGgabeaakiabgEna0oaalaaabaGaemiCaa3aaWbaaSqabeaacqWGUbGBaaGccqqGGaaicqWGHbqycqqGGaaicqWGIbGydaWgaaWcbaGaemyBa0gabeaaaOqaaiabgkHiTiGbcYgaSjabc6gaUjabdchaWbaacqGGUaGlaaa@46C7@

The first term, *m a d b*_*h*_, quantifies the number of mosquitoes expected to acquire infection from each infectious host. The second term, pn a bm−ln⁡p
 MathType@MTEF@5@5@+=feaafiart1ev1aaatCvAUfKttLearuWrP9MDH5MBPbIqV92AaeXatLxBI9gBaebbnrfifHhDYfgasaacH8akY=wiFfYdH8Gipec8Eeeu0xXdbba9frFj0=OqFfea0dXdd9vqai=hGuQ8kuc9pgc9s8qqaq=dirpe0xb9q8qiLsFr0=vr0=vr0dc8meaabaqaciaacaGaaeqabaqabeGadaaakeaadaWcaaqaaiabdchaWnaaCaaaleqabaGaemOBa4gaaOGaeeiiaaIaemyyaeMaeeiiaaIaemOyai2aaSbaaSqaaiabd2gaTbqabaaakeaacqGHsislcyGGSbaBcqGGUbGBcqWGWbaCaaaaaa@3A9C@, represents the probability that a mosquito, once infected, will transmit the agent to a susceptible host. The Ross-Macdonald expression can readily be adapted for enzootic transmission of WNV, by simply substituting birds for people. While several detailed mathematical analyses of WNV transmission have been presented [21-23], the resulting expressions for *R*_0 _offer little advantage over the Ross-Macdonald expression. As these expressions are based on next-generation matrices specified according to Diekmann et al. [24], i.e. considering the transmission from mosquito to bird and from bird back to mosquito as representing two generations, they are square roots of Ross-Macdonald-like quantities. We take the position that the full transmission cycle represents one generation and therefore prefer the notation given in expression 1. In contrast to the expression for *R*_0 _given in [21-23] the Ross-Macdonald expression does not implicitly model disease-associated mortality. However, by using an empirically measured duration of effective viremia for *d*, such process is implicitly taken into account.

The Ross-Macdonald expression implicitly assumes a constant, density-independent feeding rate of each mosquito. The lower the host density and thus the larger *m*, the more blood meals will therefore be taken on a particular host. Furthermore, reservoir incompetent hosts are not considered and thus do not divert mosquitoes from reservoir hosts. In addition, the mosquito population is assumed to be constant.

Given these assumptions, we examine the effect of extreme virulence (no survival after infection) on transmission dynamics. We will examine the validity of these assumptions and the effect of their violation. Denote the size of a local flock of reservoir competent birds *H *and the size of the associated female mosquito population *M*. Denote the sorted times at which birds that are infected with WNV die *t*_1_, *t*_2_,..., *t*_H*_, where H* ≤ *H *and *t*_H* _is the time of death of the bird that dies last. Define

R0k=MH−ka2 d pn bm bh−ln⁡p,∀ k∈(1,...,H−1),
 MathType@MTEF@5@5@+=feaafiart1ev1aaatCvAUfKttLearuWrP9MDH5MBPbIqV92AaeXatLxBI9gBaebbnrfifHhDYfgasaacH8akY=wiFfYdH8Gipec8Eeeu0xXdbba9frFj0=OqFfea0dXdd9vqai=hGuQ8kuc9pgc9s8qqaq=dirpe0xb9q8qiLsFr0=vr0=vr0dc8meaabaqaciaacaGaaeqabaqabeGadaaakeaacqWGsbGudaqhaaWcbaGaeGimaadabaGaem4AaSgaaOGaeyypa0ZaaSaaaeaadaWcaaqaaiabd2eanbqaaiabdIeaijabgkHiTiabdUgaRbaacqWGHbqydaahaaWcbeqaaiabikdaYaaakiabbccaGiabdsgaKjabbccaGiabdchaWnaaCaaaleqabaGaemOBa4gaaOGaeeiiaaIaemOyai2aaSbaaSqaaiabd2gaTbqabaGccqqGGaaicqWGIbGydaWgaaWcbaGaemiAaGgabeaaaOqaaiabgkHiTiGbcYgaSjabc6gaUjabdchaWbaacqGGSaalcqGHaiIicqqGGaaicqWGRbWAcqGHiiIZcqGGOaakcqaIXaqmcqGGSaalcqGGUaGlcqGGUaGlcqGGUaGlcqGGSaalcqWGibascqGHsislcqaIXaqmcqGGPaqkcqGGSaalaaa@5B0D@

=HH−kR00,
 MathType@MTEF@5@5@+=feaafiart1ev1aaatCvAUfKttLearuWrP9MDH5MBPbIqV92AaeXatLxBI9gBaebbnrfifHhDYfgasaacH8akY=wiFfYdH8Gipec8Eeeu0xXdbba9frFj0=OqFfea0dXdd9vqai=hGuQ8kuc9pgc9s8qqaq=dirpe0xb9q8qiLsFr0=vr0=vr0dc8meaabaqaciaacaGaaeqabaqabeGadaaakeaacqGH9aqpdaWcaaqaaiabdIeaibqaaiabdIeaijabgkHiTiabdUgaRbaacqWGsbGudaqhaaWcbaGaeGimaadabaGaeGimaadaaOGaeiilaWcaaa@3660@

where the superscript *k *in R0k
 MathType@MTEF@5@5@+=feaafiart1ev1aaatCvAUfKttLearuWrP9MDH5MBPbIqV92AaeXatLxBI9gBaebbnrfifHhDYfgasaacH8akY=wiFfYdH8Gipec8Eeeu0xXdbba9frFj0=OqFfea0dXdd9vqai=hGuQ8kuc9pgc9s8qqaq=dirpe0xb9q8qiLsFr0=vr0=vr0dc8meaabaqaciaacaGaaeqabaqabeGadaaakeaacqWGsbGudaqhaaWcbaGaeGimaadabaGaem4AaSgaaaaa@3053@ is the number of reservoir hosts that have died and R00
 MathType@MTEF@5@5@+=feaafiart1ev1aaatCvAUfKttLearuWrP9MDH5MBPbIqV92AaeXatLxBI9gBaebbnrfifHhDYfgasaacH8akY=wiFfYdH8Gipec8Eeeu0xXdbba9frFj0=OqFfea0dXdd9vqai=hGuQ8kuc9pgc9s8qqaq=dirpe0xb9q8qiLsFr0=vr0=vr0dc8meaabaqaciaacaGaaeqabaqabeGadaaakeaacqWGsbGudaqhaaWcbaGaeGimaadabaGaeGimaadaaaaa@2FE2@ = *R*_0_. Clearly, the calculations presented in expressions 2 and 3 break down when the last bird dies and the local mosquito-host ratio goes to infinity. If *k *reservoir hosts have died the basic reproductive number will be greater by the factor HH−k
 MathType@MTEF@5@5@+=feaafiart1ev1aaatCvAUfKttLearuWrP9MDH5MBPbIqV92AaeXatLxBI9gBaebbnrfifHhDYfgasaacH8akY=wiFfYdH8Gipec8Eeeu0xXdbba9frFj0=OqFfea0dXdd9vqai=hGuQ8kuc9pgc9s8qqaq=dirpe0xb9q8qiLsFr0=vr0=vr0dc8meaabaqaciaacaGaaeqabaqabeGadaaakeaadaWcaaqaaiabdIeaibqaaiabdIeaijabgkHiTiabdUgaRbaaaaa@313A@ than the original value R00
 MathType@MTEF@5@5@+=feaafiart1ev1aaatCvAUfKttLearuWrP9MDH5MBPbIqV92AaeXatLxBI9gBaebbnrfifHhDYfgasaacH8akY=wiFfYdH8Gipec8Eeeu0xXdbba9frFj0=OqFfea0dXdd9vqai=hGuQ8kuc9pgc9s8qqaq=dirpe0xb9q8qiLsFr0=vr0=vr0dc8meaabaqaciaacaGaaeqabaqabeGadaaakeaacqWGsbGudaqhaaWcbaGaeGimaadabaGaeGimaadaaaaa@2FE2@. Under the given assumptions, transmission will therefore tend to become intensified in the face of host mortality.

*R*_0 _determines the dynamics of transmission at the very beginning of an epidemic/epizootic, i.e. when all individuals are still susceptible to infection. As the epidemic progresses and increasing numbers of individuals loose their susceptibility, either due to immunity or due to current infection, transmission will become less intense than suggested by *R*_0_. The effective reproductive number *R*_*Eff *_quantifies the actual epidemic dynamics. *R*_*Eff *_is defined as *R*_0 _multiplied by the proportion susceptible *s *[17]. Hamer [25] was the first to appreciate that that quantity drives the waxing (*R*_*Eff *_> 1) and waning (*R*_*Eff *_< 1) of an epidemic. If the number of susceptibles falls below a critical level, transmission will eventually cease. As

*R*_*Eff *_= *R*_0 _*s*,

*R*_*Eff *_will be higher and closer to *R*_0 _in the absence of "herd" immunity than in its presence. Furthermore, local depletion of hosts through mortality may be epidemiologically important as an impetus for susceptible birds to immigrate and thus to facilitate local perpetuation, or for infectious mosquitoes to disperse in pursuit of feeding hosts, thus geographically spreading transmission.

### Computer simulations and sensitivity analysis

To explore the relationship between virulence and dynamic aspects of transmission, we simulated epizootics under various virulence assumptions. Specifically, we investigated seven scenarios that ranged from extremely virulent (no survival) to avirulent (100% survival). Intermediate scenarios included 10%, 25%, 50%, 75% and 90% survival. The simulations were stochastic, individual-based and time-continuous: Each mosquito and bird "behaved" according to the assumed underlying stochastic laws and was kept track of individually in the simulation. Most assumptions underlying the simulations directly derive from the Ross-Macdonald expression and have already been stated above. The most notable deviation of the simulation from the assumptions implied by the Ross-Macdonald expression relates to mosquito survival. While in the theoretical model mosquito survival is driven by an exponential "memory-less" decay process that implies absence of aging, each of our simulated mosquitoes has a unique predetermined life span (see methods section for details). Even when the mortality rate is assumed equal, our more realistically simulated mosquitoes therefore take, on average, substantially fewer blood meals after becoming infectious than would be predicted by the Ross-Macdonald model. Although recent studies have documented low levels of non-viremic and thus latency free transmission of WNV between mosquitoes [26,27], we simulated a latency period in birds according to the results from the infection experiments by Komar and colleagues [28]. Such latency period is ignored in the Ross-Macdonald expression. Reservoir hosts that survive infection were assumed to acquire permanent immunity [29]. Values for the simulation parameters were chosen on the basis of published empirical values, when available, or as used by other authors (Table 1). Most stochastic parameters were assumed to be distributed according to a Gamma distribution to ensure unimodality and a positive real domain. Life expectancy of mosquitoes was assumed to follow an exponential distribution. Mosquito demography was not modeled; rather, dying mosquitoes were immediately replaced by "new" adult females. Each simulation was run until transmission ceased. For each virulence scenario, 100 simulations were realized.

**Table 1:** Default parameter values. Parameter values and distributions used in the computer simulations.

Parameter	Symbol	Mean value	Distribution	Units	Source
Initial number of birds	*H*	20	constant	(number)	
Number of mosquitoes	*M*	1,000	constant	(number)	
Time between blood meals	1/*a*	5.0	~ Gamma(10, 0.5)	days	[21, 49]
Mosquitoes' life expectancy	-ln(*p*)	33.3	~ Exp(ln(*p*))	days	[21]
Mosquitoes' extrinsic incubation period	*n*	7.0	~ Gamma(28, 0.25)	days	[50]
Birds' time to infectiousness	-	2	~ Gamma(8, 0.25)	days	[28, 32]
Birds' duration of infectiousness	*d*	3.25	Gamma(13, 0.25)	days	[28]
Probability of bird-mosquito infection	*b*_*m*_	0.69	constant	(probability)	[51]
Probability of mosquito-bird infection	*b*_*h*_	0.74	constant	(probability)	[51]

**Figure 1:**
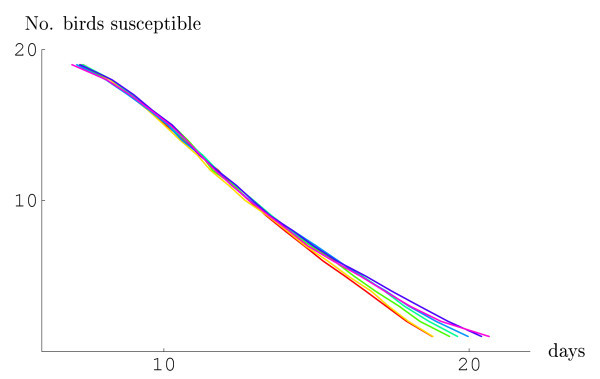
**Decline over time of susceptibility according tovarious virulence scenarios**. The curves represent birds' median ordered times to becoming infected, i.e. loosing susceptibility. The color range represents extreme virulence (100% mortality – red) to avirulence (no mortality – purple), as well as all intermediate virulence scenarios (90% – orange, 75% – light green, 50% – turquoise, 25% – light blue and 10% mortality – navy blue).

**Figure 2:**
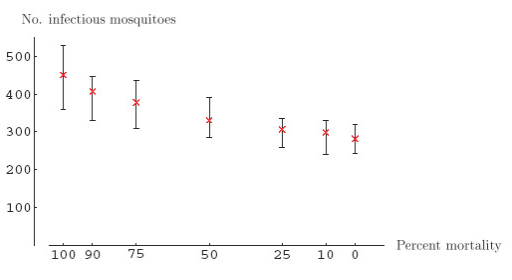
**Final distribution of the number of infectiousmosquitoes after epizootics according to various levels of virulence**. The x-axis represents the level of virulence (in terms of reservoir host mortality). The crosses represent median numbers of infectious mosquitoes at the end of the epizootic, at different levels of virulence, expressed as cumulative mortality. Error bars correspond to the 2.5th and the 97.5th percentile, respectively.

Higher virulence was consistently associated with higher "epidemic output" as measured by the number of infectious mosquitoes at the end of the epizootic (Figure 1). Except for the most extreme scenarios, the simulation results were broadly overlapping. It is worth noting that invariably all birds became infected and that therefore no susceptible birds remained, either due to death or immunity. The epizootiological dynamics, in terms of avian infections, were only moderately affected by the level of virulence (Figure 2). The mean time of the last avian infection ranged from 18.8 days after introduction of the index bird for the highest virulence to 20.7 days for the lowest virulence scenario.

Using the parameter values/means from Table 1, *R*_0 _can be calculated using the Ross-Macdonald expression:

R0=m a2 d pn bm bh−ln⁡p=1000200.22 3.25 0.977 0.74 0.690.03=89.4.
 MathType@MTEF@5@5@+=feaafiart1ev1aaatCvAUfKttLearuWrP9MDH5MBPbIqV92AaeXatLxBI9gBaebbnrfifHhDYfgasaacH8akY=wiFfYdH8Gipec8Eeeu0xXdbba9frFj0=OqFfea0dXdd9vqai=hGuQ8kuc9pgc9s8qqaq=dirpe0xb9q8qiLsFr0=vr0=vr0dc8meaabaqaciaacaGaaeqabaqabeGadaaakeaafaqadeWabaaabaGaemOuai1aaSbaaSqaaiabicdaWaqabaGccqGH9aqpdaWcaaqaaiabd2gaTjabbccaGiabdggaHnaaCaaaleqabaGaeGOmaidaaOGaeeiiaaIaemizaqMaeeiiaaIaemiCaa3aaWbaaSqabeaacqWGUbGBaaGccqqGGaaicqWGIbGydaWgaaWcbaGaemyBa0gabeaakiabbccaGiabdkgaInaaBaaaleaacqWGObaAaeqaaaGcbaGaeyOeI0IagiiBaWMaeiOBa4MaemiCaahaaaqaaiabg2da9maalaaabaWaaSaaaeaacqaIXaqmcqaIWaamcqaIWaamcqaIWaamaeaacqaIYaGmcqaIWaamaaGaeGimaaJaeiOla4IaeGOmaiZaaWbaaSqabeaacqaIYaGmaaGccqqGGaaicqaIZaWmcqGGUaGlcqaIYaGmcqaI1aqncqqGGaaicqaIWaamcqGGUaGlcqaI5aqocqaI3aWndaahaaWcbeqaaiabiEda3aaakiabbccaGiabicdaWiabc6caUiabiEda3iabisda0iabbccaGiabicdaWiabc6caUiabiAda2iabiMda5aqaaiabicdaWiabc6caUiabicdaWiabiodaZaaaaeaacqGH9aqpcqaI4aaocqaI5aqocqGGUaGlcqaI0aancqGGUaGlaaaaaa@6EA4@

From the simulations, we can also calculate an empirical value of *R*_0 _and obtain R^
 MathType@MTEF@5@5@+=feaafiart1ev1aaatCvAUfKttLearuWrP9MDH5MBPbIqV92AaeXatLxBI9gBaebbnrfifHhDYfgasaacH8akY=wiFfYdH8Gipec8Eeeu0xXdbba9frFj0=OqFfea0dXdd9vqai=hGuQ8kuc9pgc9s8qqaq=dirpe0xb9q8qiLsFr0=vr0=vr0dc8meaabaqaciaacaGaaeqabaqabeGadaaakeaacuWGsbGugaqcaaaa@2DE9@_0 _= 28.3 from the mean of observed values (2.5^*th *^percentile:8; 97.5^*th *^percentile: 48) (see Methods section for details). The difference between the theoretical and the empirical value is attributable to a higher number of mosquitoes that are expected from the Ross-Macdonald expression to become infectious from one viremic bird compared to the observed mean value (18.2 vs. 15.0), as well as to the much higher number of blood meals a mosquito is expected to take after becoming infectious, compared to observed values (6.67 vs. 1.58). The discrepancy between these predictions is fully explained by the way mosquito survival is modeled. Not surprisingly, given the high value of *R*_0_, all simulations led to large outbreaks.

### Deviations from assumptions and sensitivity analysis

**Figure 3:**
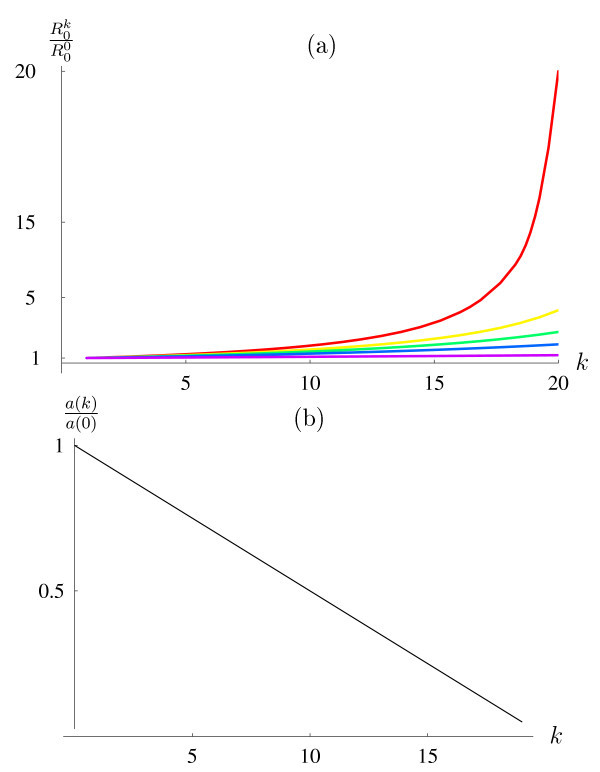
**Sensitivity analysis**. (a) The factor by which R0k
 MathType@MTEF@5@5@+=feaafiart1ev1aaatCvAUfKttLearuWrP9MDH5MBPbIqV92AaeXatLxBI9gBaebbnrfifHhDYfgasaacH8akY=wiFfYdH8Gipec8Eeeu0xXdbba9frFj0=OqFfea0dXdd9vqai=hGuQ8kuc9pgc9s8qqaq=dirpe0xb9q8qiLsFr0=vr0=vr0dc8meaabaqaciaacaGaaeqabaqabeGadaaakeaacqWGsbGudaqhaaWcbaGaeGimaadabaGaem4AaSgaaaaa@3053@ increases compared to R00
 MathType@MTEF@5@5@+=feaafiart1ev1aaatCvAUfKttLearuWrP9MDH5MBPbIqV92AaeXatLxBI9gBaebbnrfifHhDYfgasaacH8akY=wiFfYdH8Gipec8Eeeu0xXdbba9frFj0=OqFfea0dXdd9vqai=hGuQ8kuc9pgc9s8qqaq=dirpe0xb9q8qiLsFr0=vr0=vr0dc8meaabaqaciaacaGaaeqabaqabeGadaaakeaacqWGsbGudaqhaaWcbaGaeGimaadabaGaeGimaadaaaaa@2FE2@ as a function of cumulative host mortality *k *(x-axis), in the presence of various "numbers" of alternate hosts. These "numbers" represent attractiveness of alternative hosts relative to 20 reservoir hosts. The presence of 20 alternative hosts, for example, corresponds to equal attractiveness of the alternative hosts when all reservoir hosts are still alive. The red curve corresponds to no alternative hosts available. The other curves, shown in yellow, green, blue and purple represent 5, 10, 20, and 100 alternative hosts available, respectively. (b) This graph shows the decrease in the biting rate *a *(*k*) relative to the initial biting rate *a*(0) (y-axis) as a function of cumulative host mortality *k *(x-axis) for which the number of mosquito bites per host remain unchanged, i.e. R00
 MathType@MTEF@5@5@+=feaafiart1ev1aaatCvAUfKttLearuWrP9MDH5MBPbIqV92AaeXatLxBI9gBaebbnrfifHhDYfgasaacH8akY=wiFfYdH8Gipec8Eeeu0xXdbba9frFj0=OqFfea0dXdd9vqai=hGuQ8kuc9pgc9s8qqaq=dirpe0xb9q8qiLsFr0=vr0=vr0dc8meaabaqaciaacaGaaeqabaqabeGadaaakeaacqWGsbGudaqhaaWcbaGaeGimaadabaGaeGimaadaaaaa@2FE2@ = R0n
 MathType@MTEF@5@5@+=feaafiart1ev1aaatCvAUfKttLearuWrP9MDH5MBPbIqV92AaeXatLxBI9gBaebbnrfifHhDYfgasaacH8akY=wiFfYdH8Gipec8Eeeu0xXdbba9frFj0=OqFfea0dXdd9vqai=hGuQ8kuc9pgc9s8qqaq=dirpe0xb9q8qiLsFr0=vr0=vr0dc8meaabaqaciaacaGaaeqabaqabeGadaaakeaacqWGsbGudaqhaaWcbaGaeGimaadabaGaemOBa4gaaaaa@3059@. Points above the curve, but below 1 represent biting rates that decrease with increasing mosquito-host rates, but will still result in a net increase of mosquito bites per host.

The assumption that only reservoir competent hosts are locally available to mosquitoes is hardly realistic. The local presence of reservoir incompetent hosts may profoundly affect the resulting epidemiologic dynamics ("zooprophylactic" effect [30]). To examine the potential impact of reservoir incompetent hosts on WNV transmission dynamics, we explored the effect of extreme virulence on *R*_0 _in the presence of various densities of alternative hosts (Figure 3a). As expected, the transmission-boosting effect of host mortality is mitigated by the presence of alternative hosts. That mitigation is quite powerful, even when alternative hosts are scarce. Accordingly, R0k
 MathType@MTEF@5@5@+=feaafiart1ev1aaatCvAUfKttLearuWrP9MDH5MBPbIqV92AaeXatLxBI9gBaebbnrfifHhDYfgasaacH8akY=wiFfYdH8Gipec8Eeeu0xXdbba9frFj0=OqFfea0dXdd9vqai=hGuQ8kuc9pgc9s8qqaq=dirpe0xb9q8qiLsFr0=vr0=vr0dc8meaabaqaciaacaGaaeqabaqabeGadaaakeaacqWGsbGudaqhaaWcbaGaeGimaadabaGaem4AaSgaaaaa@3053@ will only substantially increase in relation to R00
 MathType@MTEF@5@5@+=feaafiart1ev1aaatCvAUfKttLearuWrP9MDH5MBPbIqV92AaeXatLxBI9gBaebbnrfifHhDYfgasaacH8akY=wiFfYdH8Gipec8Eeeu0xXdbba9frFj0=OqFfea0dXdd9vqai=hGuQ8kuc9pgc9s8qqaq=dirpe0xb9q8qiLsFr0=vr0=vr0dc8meaabaqaciaacaGaaeqabaqabeGadaaakeaacqWGsbGudaqhaaWcbaGaeGimaadabaGaeGimaadaaaaa@2FE2@ as a function of *k *(reservoir host mortality) when most blood meals will be taken on reservoir hosts, regardless of how many reservoir hosts have died.

We have further assumed that mosquitoes blood feed at a constant, density independent rate *a*. Experimental evidence, however, suggests that an increase in the mosquito-host ratio does not necessarily translate into a higher feeding rate on a particular host [31]. Clearly, if reservoir hosts are not blood-fed on at a higher density when the mosquito-host ratio increases, then host mortality will not affect transmission dynamics. This would be the case when the feeding rate of each mosquito decreases enough to compensate for the increasing mosquito-host ratio. To derive the threshold of the biting rate as a function of the mosquito-host ratio, above which the feeding rate per host still increases, let *a*(*k*) be the biting rate when *k *hosts have perished, for *k *∈ (0, 1,..., *H *- 1). When *k *hosts have perished, each reservoir host will thus receive mosquito bites at the rate *a*(*k*)MH−k
 MathType@MTEF@5@5@+=feaafiart1ev1aaatCvAUfKttLearuWrP9MDH5MBPbIqV92AaeXatLxBI9gBaebbnrfifHhDYfgasaacH8akY=wiFfYdH8Gipec8Eeeu0xXdbba9frFj0=OqFfea0dXdd9vqai=hGuQ8kuc9pgc9s8qqaq=dirpe0xb9q8qiLsFr0=vr0=vr0dc8meaabaqaciaacaGaaeqabaqabeGadaaakeaadaWcaaqaaiabd2eanbqaaiabdIeaijabgkHiTiabdUgaRbaaaaa@3144@. If

a(k)MH−k=a(0)MH, for k∈(0,1,...,H−1),
 MathType@MTEF@5@5@+=feaafiart1ev1aaatCvAUfKttLearuWrP9MDH5MBPbIqV92AaeXatLxBI9gBaebbnrfifHhDYfgasaacH8akY=wiFfYdH8Gipec8Eeeu0xXdbba9frFj0=OqFfea0dXdd9vqai=hGuQ8kuc9pgc9s8qqaq=dirpe0xb9q8qiLsFr0=vr0=vr0dc8meaabaqaciaacaGaaeqabaqabeGadaaakeaacqWGHbqycqGGOaakcqWGRbWAcqGGPaqkdaWcaaqaaiabd2eanbqaaiabdIeaijabgkHiTiabdUgaRbaacqGH9aqpcqWGHbqycqGGOaakcqaIWaamcqGGPaqkdaWcaaqaaiabd2eanbqaaiabdIeaibaacqGGSaalcqqGGaaicqqGMbGzcqqGVbWBcqqGYbGCcqqGGaaicqWGRbWAcqGHiiIZcqGGOaakcqaIWaamcqGGSaalcqaIXaqmcqGGSaalcqGGUaGlcqGGUaGlcqGGUaGlcqGGSaalcqWGibascqGHsislcqaIXaqmcqGGPaqkcqGGSaalaaa@5303@

the rate of mosquitoes feeding per host will remain unchanged between before the first host death and the *k*th death among reservoir hosts. Therefore,

a(k)=a(0)MHMH−k=a(0)H−kH
 MathType@MTEF@5@5@+=feaafiart1ev1aaatCvAUfKttLearuWrP9MDH5MBPbIqV92AaeXatLxBI9gBaebbnrfifHhDYfgasaacH8akY=wiFfYdH8Gipec8Eeeu0xXdbba9frFj0=OqFfea0dXdd9vqai=hGuQ8kuc9pgc9s8qqaq=dirpe0xb9q8qiLsFr0=vr0=vr0dc8meaabaqaciaacaGaaeqabaqabeGadaaakeaafaqadeGabaaabaGaemyyaeMaeiikaGIaem4AaSMaeiykaKIaeyypa0JaemyyaeMaeiikaGIaeGimaaJaeiykaKYaaSaaaeaadaWcaaqaaiabd2eanbqaaiabdIeaibaaaeaadaWcaaqaaiabd2eanbqaaiabdIeaijabgkHiTiabdUgaRbaaaaaabaGaeyypa0JaemyyaeMaeiikaGIaeGimaaJaeiykaKYaaSaaaeaacqWGibascqGHsislcqWGRbWAaeaacqWGibasaaaaaaaa@467B@

defines the linear threshold function along which the number of mosquito bites per host are constant (Figure 3b). Even if the feeding rate declines with an increasing relative mosquito density, transmission will increase with host mortality, as long as the feeding rate remains above that threshold function, i.e *a*(*k*) > *a*(0)H−kH
 MathType@MTEF@5@5@+=feaafiart1ev1aaatCvAUfKttLearuWrP9MDH5MBPbIqV92AaeXatLxBI9gBaebbnrfifHhDYfgasaacH8akY=wiFfYdH8Gipec8Eeeu0xXdbba9frFj0=OqFfea0dXdd9vqai=hGuQ8kuc9pgc9s8qqaq=dirpe0xb9q8qiLsFr0=vr0=vr0dc8meaabaqaciaacaGaaeqabaqabeGadaaakeaadaWcaaqaaiabdIeaijabgkHiTiabdUgaRbqaaiabdIeaibaaaaa@313A@.

## Discussion

The factors leading to the rapid and consistent spread of WNV through North America are not well understood. Clearly, efficient replication of the virus, vector competence, and reservoir competence are prerequisites for transmission and perpetuation of WNV [32] as well as for other mosquito-borne viruses. Mosquito behavior [33] and vector mosquito species composition [34] have been speculated to contribute to the frequent transmission of WNV to people. Here, we examine the possibility that, under certain conditions and independently of the mentioned factors, reservoir mortality might have contributed to the rapid spread of WNV in North America.

By inspection of the Ross-Macdonald expression we find that *R*_0 _increases with reservoir mortality due to the increasing mosquito-host ratio. Wonham et al. previously noted that "reducing crow densities would be expected to enhance disease transmission [of WNV], because *R *scales positively with the mosquito-bird ratio" [21]. Yet, the potential implications of this epidemiologic mechanism remained unexplored. Here, we have illustrated this "concentration effect" by a simulation experiment that confirms that substantially more mosquitoes may become infected in the course of an epizootic that involves a highly virulent agent compared to an agent of low virulence. The effect of the level of virulence on the dynamics of the avian epizootic that became apparent in the simulation study, on the other hand, was moderate, due to the high starting value of *R*_0 _that virtually guaranteed universal infection. Cruz-Pacheco and colleagues [23] estimate *R*_0 _for American crows to be 21, which is much lower than our Ross-Macdonald estimate. However, the comparison between *R*_0 _estimates is determined by assumptions about parameters and therefore not very meaningful. The difference between their and our estimate is fully accounted for by their ten times lower mosquito-host ratio, a higher mosquito mortality, a shorter effective duration of viremia and the lack of an extrinsic incubation period.

Our simulation experiments did not address that effect of virulence that may emerge as the most important one: if all local reservoir hosts survive and continue to serve as feeding hosts to vector mosquitoes, then most potentially infectious mosquito bites that originated in the epizootic would be "wasted" on immune hosts. In that cases, WNV might fail to perpetuate locally. If, on the other hand, susceptible birds would immigrate to the site and take the place of perished birds, the level of susceptibility might remain sufficiently high for local perpetuation. Finally, if the local host die-off would force mosquitoes to disperse, geographic spread of WNV transmission might result. This might also contribute to zoonotic transmission and consequently to human disease.

The argument regarding the potential epidemiological effect of high virulence on WNV transmission, perpetuation and spread is speculative and, like the simulation experiments, depends on a set of specific assumptions. First, we assume that vector mosquitoes (such as *Cx. pipiens*) and reservoir hosts are closely and spatially stably associated. We are not aware of direct empirical evidence for such association. Several studies, however, have addressed different aspects of this premise. Most important and instructive is the study by Ward et al. [35]. By following 31 radio-tagged American crows over WNV transmission seasons, these authors found that the surveyed crows always roosted communally, but did not use constant roosting sites. Rather, their roosting sites were, on average, over one kilometer distant from previous night's roosting site. Clearly, this indicates that for the species most likely to exhibit extreme mortality due to WNV this important premise may not hold. On the other hand, the average number of roosts used in a five-day period was below two, indicating that a limited numbers of roosts was used. While mortality would still lead to a concentration effect, because fewer hosts would be available to vector mosquitoes than would have been otherwise, the epidemiological effect of this roosting behavior is difficult to predict. If a flock of reservoir birds were consistently absent from a roost when infectious mosquitoes would tend to feed, a lower *R*_0 _would result than predicted by the Ross-Macdonald expression. The opposite might be true if roost occupancy and mosquito feeding were synchronized. On the other hand, if viremic birds behaved in a way that would preclude them from being exposed to that concentration effect, the increased *R*_0 _would remain inconsequential. The mentioned study indicated that viremic crows tended to range even further than healthy ones, at least until shortly before their death [35]. We have insufficient information to epidemiologically evaluate this observation. Anderson et al. [36] found that *Cx. pipiens *were not only more than three times as abundant in the tree canopy as at ground level, but prevalence of WNV infection in mosquitoes was also higher in the tree canopy than at ground level. This finding suggests that populations of vector mosquitoes are spatially structured and associated with sites where certain reservoir hosts are likely to roost. A study by Drummond et al. [37], on the other hand, did not find a greater abundance of *Cx. pipiens *in the canopy compared to the ground level. Overall, the first assumption is likely not entirely realistic. Second, for host mortality to substantially affect transmission dynamics, alternative feeding hosts must not be readily available. For malaria, which has no non-human reservoir, the presence of alternative sources of blood has long been known to have a prophylactic effect on transmission. This effect has been termed "zooprophylaxis" [30] because, in the case of malaria, those alternative hosts are always non-human vertebrates. Recent blood meal analyses of *Cx. pipiens *as well as of other potential vector mosquitoes reveal a somewhat contradictory picture regarding the host species these mosquitoes tend to feed on. Molaei et al. [38], for example, found that more than 93 % of analyzed *Cx. pipiens *(N = 204) and all *Cx. restuans *(N = 30) contained avian blood. Another study [39] found that the proportion of avian blood meals varied for both species by location. In one location the corresponding proportions were as high as 84% (N = 19) for *Cx. pipiens *and 80% (N = 10) for *Cx. restuans*, while in a different location, these proportions were as low 35% (N = 190) and 52% (N = 29), respectively. A partial reconciliation of these results may be a seasonal shift in host preferences, as was documented for *Cx. pipiens *over a summer, starting with a preference for birds, but developing a stronger tendency to feed on mammals later in the season [33]. Under certain circumstances, potential vector mosquitoes of WNV therefore appear to feed mainly on birds. It is, however, important to note that some avian species appear to be relatively poor reservoirs, while others lack reservoir competency altogether [28,40]. Third, for the proposed mechanism to be locally effective, a substantial proportion of birds infected with WNV would be required to perish. North American isolates of WNV are extremely virulent for American crows (*Corvus brachyrhynchos*), at least in the laboratory [28,32]. Some field observations support extraordinary virulence for American crows [13,41]. Some studies, however, have found a seroprevalence of antibodies to WNV in crows between 10 and 50% [42,43] that is compatible with substantial survival of these birds. Yet, it is important to note, that even with very high mortality, substantial proportions of WNV immune might be recorded, because the selective die-off of birds vulnerable to fatal WNV infection. However, a majority of the bird-derived blood meals appears to be taken on American robins (*Turdus migratorius*) [38,39,44], a species which is reservoir competent, but appears to be relatively resistant to WNV infection [28]. These observations are not in support of the scenario that American crows, or another species suffering extreme mortality after WNV infection, play a crucial role in the current epidemiology of WNV. Clearly, American crows are not required for WNV to perpetuate. On the other hand, the virtual absence of American crows among the species identified in these studies as sources for mosquito blood meals could in part be due to population decline of that species. In some areas, American crow populations declined by 90% during the first three years of the epizootic [13]. During the first major epizootic in California in 2004, Reisen et al. [45] found that WNV epizootics were significantly spatially associated with American crow roosts and crow mortality. This suggest that, at least in the early stages of WNV emergence, American crows may play an important epidemiologic role. Finally, reservoir host mortality will only translate into a higher *R*_0 _if the increase in the mosquito-host ratio results in an increase in the average number of blood meals per bird. However, mosquito density may interfere with feeding success. Edman et al. [31] observed that, in some avian species (for example cattle egret, *Bubulcus ibis*) anti-mosquito activity left the number of mosquitoes feeding successfully per bird more or less unchanged, even when mosquito numbers increased by orders of magnitude. On the other hand, defensive behavior of a bird may increase the frequency of host contact [46]. How this would affect the probability of transmission from the reservoir host to the vector mosquito (*b*_*h*_) and vice versa (*b*_*m*_) remains to be determined experimentally.

Reservoir mortality certainly is not a prerequisite for perpetuation of WNV. Under certain conditions, however, reservoir mortality might enhance transmission and might have contributed to the epidemiologic vigor of WNV transmission in North America. The factors responsible for the observed differences in bird mortality between the Old and the New World remain to be identified. It could be that the avifauna of North America is more susceptible to widespread and large epizootics for reasons of species richness [47] or due to population genetic factors [48]. Conversely, the strain of WNV that was introduced into North America could be more virulent than those circulating in Eurasia and Africa. The avian mortality that was seen in the WNV outbreak in Israel of 1998 [9,10] would appear to favor the latter explanation. Overall, it is unclear whether the increase in the mosquito-host ratio and lack of herd immunity contributes substantially to the ongoing WNV epizootic in North America. However, we believe that this possibility should be seriously considered and subject to empirical examination.

## Conclusion

Since its emergence in North America, WNV has become the most important cause of mosquito-transmitted disease on that continent. In contrast to the situation in the Old World, widespread mortality among wild birds, especially corvids, has accompanied this disease emergence. We have examined specific conditions under which host mortality may be a pivotal factor in the emergence of WNV in North America and propose that this mechanism is worth empirical examination.

## Methods

### Computer simulations

The simulations presented here were based on twenty fully susceptible birds and 1,000 female mosquitoes that "behaved" according to stochastic laws. Values for all parameters used in these simulation experiments are shown in Table 1. The mosquito population was held constant by immediately replacing a dying mosquito by another mosquito. The simulation was implemented by creating, through simulation, a list of mosquitoes with particular life-events, i.e. blood-feeding times and times of death. The population process was assumed to be independent of the infection process, i.e. feeding and death in mosquitoes were assumed not to be affected by viral infection. For computational efficiency, members of the list that represented dying mosquitoes were eliminated because they were epidemiologically inconsequential. Simultaneously, a list of birds with associated durations of the latency and viremic period was created. These times would only be realized in the case of infection. Bird mortality was modeled stochastically with a scenario-specific probability of surviving infection that would occur at the end of the infectious period. All simulations began with an identical list of mosquitoes with associated life histories as well as an identical list of birds. According to this set-up, the feeding and death processes of mosquitoes were deterministic and thus identical for all simulations, while the infection process was stochastic. A simulated local epidemic started with one viremic bird with a fixed viremic period of 3.25 days (mean duration of infectiousness). Mosquitoes randomly "chose" among available birds. If a susceptible mosquito fed on a viremic bird, it became infected with probability *b*_*h *_and infectious if it survived the extrinsic incubation period. Once bitten by an infectious mosquito, a bird entered a latency period of infection with probability *b*_*m*_. After the end of the latency period, a bird became infectious for a time that was predetermined in each bird. A bird then either died with probability given by the virulence scenario or became immune for the rest of its life. For each scenario, 100 simulations were realized. The simulations were implemented in Mathematica (Wolfram Research, Inc). The Mathematica code for the simulations can be obtained from the authors (IMF).

To calculate the empirical distribution of *R*_0_, we kept track of all mosquitoes infected by the index bird, counted the infectious blood meals taken by them after the extrinsic incubation period and multiplied this number by the probability that a mosquito transmitted infection (*b*_*m*_). As this process was not affected by the level of virulence (no mortality until the end of the infectious period of the index bird) all simulations from all virulence settings were used simultaneously for this calculation. The 2.5th and 97.5th percentile were calculated from all 700 realizations of *R*_0_.

## Authors' contributions

IMF formulated the concept that led to this model, conducted the computer simulations and prepared the first draft of this report. AS helped develop the concept and participated in the writing of this report. Many comments of four anonymous reviewers, under the sensible direction of Dr. P. S. Agutter, Editor-in-Chief, helped improve an earlier version of the manuscript.
